# Designing Digital Health Technology to Support Patients Before and After Bariatric Surgery: Qualitative Study Exploring Patient Desires, Suggestions, and Reflections to Support Lifestyle Behavior Change

**DOI:** 10.2196/29782

**Published:** 2022-03-04

**Authors:** Anna Robinson, Andrew Husband, Robert Slight, Sarah P Slight

**Affiliations:** 1 School of Pharmacy Newcastle University Newcastle upon Tyne United Kingdom; 2 Population Health Sciences Institute Newcastle University Newcastle upon Tyne United Kingdom; 3 Newcastle upon Tyne Hospitals NHS Foundation Trust Newcastle upon Tyne United Kingdom

**Keywords:** digital technology, eHealth, mHealth, bariatric surgery, behavior change, qualitative research, co-design, perioperative care, mobile phone

## Abstract

**Background:**

A patient’s capability, motivation, and opportunity to change their lifestyle are determinants of successful outcomes following bariatric surgery. Lifestyle changes before and after surgery, including improved dietary intake and physical activity levels, have been associated with greater postsurgical weight loss and improved long-term health. Integrating patient-centered digital technologies within the bariatric surgical pathway could form part of an innovative strategy to promote and sustain healthier behaviors, and provide holistic patient support, to improve surgical success. Previous research focused on implementing digital technologies and measuring effectiveness in surgical cohorts. However, there is limited work concerning the desires, suggestions, and reflections of patients undergoing bariatric surgery. This qualitative investigation explores patients’ perspectives on technology features that would support behavior changes during the pre- and postoperative periods, to potentially maintain long-term healthy lifestyles following surgery.

**Objective:**

This study aims to understand how digital technologies can be used to support patient care during the perioperative journey to improve weight loss outcomes and surgical success, focusing on *what* patients want from digital technologies, *how* they want to use them, and *when* they would be of most benefit during their surgical journey.

**Methods:**

Patients attending bariatric surgery clinics in one hospital in the North of England were invited to participate. Semistructured interviews were conducted with purposively sampled pre- and postoperative patients to discuss lifestyle changes and the use of digital technologies to complement their care. The interviews were audio recorded and transcribed verbatim. Reflexive thematic analysis enabled the development of themes from the data. Ethical approval was obtained from the National Health Service Health Research Authority.

**Results:**

A total of 20 patients were interviewed (preoperative phase: 40% (8/20); postoperative phase: 60% (12/20). A total of 4 overarching themes were developed and related to the optimization of technology functionality. These centered on providing tailored content and support; facilitating self-monitoring and goal setting; delivering information in an accessible, trusted, and usable manner; and meeting patient information-seeking and engagement needs during the surgical pathway. Functionalities that delivered personalized feedback and postoperative follow-up were considered beneficial. Individualized goal setting functionality could support a generation of digitally engaged patients with bariatric conditions as working toward achievable targets was deemed an effective strategy for motivating behavior change. The creation of digital *package of care* checklists between patients and clinicians was a novel finding from this study.

**Conclusions:**

Perceptions of patients undergoing bariatric surgery validated the integration of digital technologies within the surgical pathway, offering enhanced connectedness and support. Recommendations are made relating to the design, content, and functionality of digital interventions to best address the needs of this cohort. These findings have the potential to influence the co-design and integration of person-centered, perioperative technologies.

## Introduction

### Background

Obesity is a growing global pandemic [1-3]. Weight loss surgery (bariatric surgery) is regarded as the most effective method for long-term weight loss [4]. Despite an increase in the number of bariatric procedures over the past few years, recent literature has suggested that surgery is still an underused treatment option, with the number of American adults choosing surgery being approximately 1% [5,6]. Despite the promising weight loss outcomes following surgery, patients can experience challenges beyond the procedure itself in their bid for surgical *success* [7]. These include facing social pressures and stigma related to surgery [8]; psychological impacts, including negative body image and depression [9]; and adjusting to postoperative lifestyle recommendations to reduce weight regain [10].

A patient’s capability, motivation, and opportunity to change their lifestyle are significant determinants of successful outcomes following bariatric surgery [11,12]. Healthier lifestyle changes before and after surgery, including improved dietary intake and physical activity levels, have been shown to contribute to greater postsurgical weight loss [13,14], maintenance of weight loss [15], and better overall long-term health [16]. However, previous literature has demonstrated the various challenges that clinicians may face when supporting changes of this nature for this surgical patient cohort, particularly on a long-term basis [17,18]. Attendance at postsurgical follow-up care [19,20], engagement with behavioral appointments and support groups [21,22], and the impacts of travel distance to clinic appointments are some of the previously examined factors associated with poorer outcomes following surgery [17,23]. Digital technologies may pose as a promising alternative avenue for the provision of surgical patient support, which could be offered remotely, without the need for in-person attendance [24]. In particular, digital technologies offer an ability to provide scalable support which may prove useful on a wide scale [24,25]. Currently, little is known about the optimal way to design, deliver, and implement digital health technologies for this unique surgical patient cohort; this study seeks to provide further insights and has adopted a patient-informed and patient-centered approach to do so [14,26].

Digital health technologies (such as mobile phone apps, tailored web platforms, and wearable activity trackers) provide promising opportunities for connected patient care. They provide education and information in an easily accessible and patient-friendly manner [25,27,28] and encourage patients to become active participants in their own care [29,30]. Studies have acknowledged patients’ receptiveness toward using digital technologies to complement the care pathways of other surgical procedures, which has resulted in successful behavior change, improved recovery time, and reduced length of stay in hospital [31-33]. In the bariatric surgery literature, recent studies have reported how telemedicine and digitally supported care have been well received by patients [34] and have potentially improved postoperative clinic attendance and patient engagement with surgical care [25,35,36]. Using digital technologies within the bariatric surgical pathway, both pre- and postoperatively, could form part of a remote strategy to deliver support and behavior change advice to patients.

Existing literature has suggested that collaborative approaches in medicine, between patients and clinicians, can result in improved patient engagement, trust, and satisfaction, and improve intended health-related outcomes [37-39]. Cocreation and user-centered, experience-based co-design approaches are being researched and implemented in other areas of health care, with the goal of improving patient-focused care [40,41]. Many studies have focused on implementing digital technologies and measuring their effectiveness in various medical and surgical cohorts [42,43]. A recent study by Korpershoek et al [44] using user-centered design approaches supported patient self-management of chronic obstructive pulmonary disease and that by Solem et al [45] designed and developed an electronic health pain management intervention for those affected by chronic pain. Similarly, a recent study by Paton et al [46] demonstrated how predictive human–computer interaction modeling could be integrated into user-centered design approaches to improve health intervention usability and safety. However, there is a paucity of patient-centered research specifically concerning the desires, suggestions, and reflections of patients undergoing bariatric surgery. This warrants further investigation to develop useful and effective digital support strategies for this patient population, with user-centered design being one possible strategy to adopt to understand how patients undergoing bariatric surgery *want* to be supported.

### Objectives

This qualitative study aims to understand how digital technologies could be used to better support patients across the wider perioperative pathway, covering pre- and postoperative time points, with the overall rationale of improving weight loss outcomes and, therefore, surgical success. Specifically, our key research questions were as follows: *What* do patients want from digital health technologies, *How* do they want to use them, and *When* would they be of most benefit during their surgical journey?

## Methods

### Participant Recruitment and Sampling

According to the Enhancing the QUAlity and Transparency Of health Research guidelines, the COREQ (Consolidated Criteria for Reporting Qualitative Research) checklist was followed for this study (Multimedia Appendix 1) [47]. Patients attending bariatric surgery clinics within a large teaching hospital in the North of England were invited to participate in this study. This included both pre- and postoperative patients who attended their outpatient appointments, as their experiences and perspectives on using digital health technologies may differ. No previous relationship was established between the researcher and participants before study commencement or recruitment. All participants were provided with an information sheet detailing the purpose and aims of the study during their appointment with the surgeon. Written informed consent was obtained before conducting the interviews. To be included in the study, patients had to be aged >18 years; recently undergone (within the last 2 years, as per the 2-year National Health Service [NHS] bariatric surgery follow-up guidelines) or planned to undergo (ie, those who are under the care of the multidisciplinary team and are awaiting a surgery date) bariatric surgery at the specific hospital trust [48]; medically stable (not affected by an acute decline in health away from baseline); and able to participate in an interview, communicate in English, and have the capacity to consent to taking part in the study. It was deemed important that participants with a range of experiences of and opinions on digital technologies were included in this study to showcase representative views reflective of those of typical patient cohorts; thus, there was no specification to the level of current or previous digital technology use to take part in this study. However, details of the frequency of technology use were collected to aid in the interpretation of the results (Table 1). Purposive sampling techniques were used to recruit a wide and representative sample of patients undergoing bariatric surgery within the region. This also meant that the sample of participants included in this study represented a mixture of ages, men and women, and included participants who were at various stages within their pre- and postoperative journeys (ranging from 1 week before surgery to 24 months after surgery).

**Table 1:** Participant characteristics (N=20).

Participant number	Sex	Age (years)	Participant ethnicity (self-reported verbatim from participant interviews)	Surgical procedure	Phase	Time since surgery (exact) or time until surgery (approximate^a^)	Reported level of digital technology use^b^ at the time of interview
1	Female	29	“English”	Gastric bypass	Postoperative	24 months	Daily
2	Female	55	“White British”	Sleeve gastrectomy	Postoperative	12 months	Daily
3	Female	54	“Pakistani Asian”	Gastric band	Postoperative	18 months	Daily
4	Female	50	“British”	Sleeve gastrectomy	Postoperative	24 months	Daily
5	Male	46	“British”	Undecided	Preoperative	6 weeks	Every other day
6	Female	52	“British”	Gastric bypass	Postoperative	9 months	Daily
7	Female	61	“English”	Gastric bypass	Postoperative	4 months	Never
8	Male	51	“British”	Gastric band	Postoperative	24 months	Daily
9	Female	39	“White British”	Sleeve gastrectomy	Preoperative	2 weeks	Daily
10	Male	40	“Asian”	Gastric bypass	Preoperative	8 weeks	Daily
11	Female	31	“British”	Gastric bypass	Postoperative	24 months	Daily
12	Female	51	“British”	Gastric bypass	Postoperative	24 months	Daily
13	Female	58	“White British”	Gastric bypass	Postoperative	24 months	Daily
14	Female	50	“White British”	Gastric bypass	Preoperative	1 week	Daily
15	Female	59	“English”	Gastric bypass	Postoperative	24 months	Every other day
16	Female	29	“Pakistani”	Gastric bypass	Postoperative	12 months	Daily
17	Male	26	“Asian”	Sleeve gastrectomy	Preoperative	8 weeks	Daily
18	Female	35	“British”	Gastric band	Preoperative	4 weeks	Daily
19	Male	50	“White British”	Undecided	Preoperative	2 weeks	Daily
20	Female	52	“British Indian”	Gastric bypass	Preoperative	4 weeks	Daily

^a^Time until surgery, approximate: given the implications of the COVID-19 pandemic, some surgery dates may have been delayed.

^b^Reported level of digital technology use: reported by participants in response to the question *How often do you use the internet or use apps on a smartphone?*

### Semistructured Interview

Between February and March 2020, in-depth semistructured interviews were conducted by a researcher (AR, a female doctoral researcher with experience in qualitative research). All participants chose to be interviewed in the hospital in a confidential surgery clinic room, at a time convenient for them; only the participant and researcher were present. Interviews were conducted until theoretical data saturation was reached, that is, upon author consensus that subsequent interviews yielded no new information. Instead, the authors observed mounting instances of the same codes, as described by Urquhart et al [49], Birks and Mills [50], and Olshansky and de Chesnay [51], and deemed that theoretical data saturation had been achieved. A semistructured interview schedule (topic guide), which formed the basis of all participant interviews, was developed based on 3 pilot interviews, existing studies on digital health technologies in this cohort [27,28,52,53], and systematic reviews of the literature by the research team [24,25]. Participant interviews included questions to elicit spontaneous discussions around their surgical experience, awareness of health and lifestyle behavior change (eg, physical activity, smoking cessation, alcohol intake, and dietary intake), patient physical and psychological support requirements, their perspectives on digital health technologies, and previous technology use (Item 2: Topic guide in Multimedia Appendix 1).

### Data Analysis

Semistructured interviews were audio recorded and transcribed verbatim by a researcher (AR). All data were anonymized at the point of transcription. Participants did not provide comment on the transcript or feedback on results. Each interview was transcribed and analyzed before conducting the next interview. Reflexive thematic analysis, as defined by Braun and Clarke [54,55], was performed by 2 researchers (AR and AKH). Transcribing the audio files and reading and rereading the interview transcripts ensured data familiarization. Significant phrases and sections of transcripts were identified and coded with initial descriptive codes; these were then sorted and clustered into common coding patterns, which enabled the development of analytic themes (derived from the data). Working iteratively and reflexively, the themes were continuously reviewed and refined until they were coherent and distinctive [54]. Reflexive analysis was performed through discussion between the 2 researchers (AR and AKH) and, if agreement was not reached, by consensus with the wider team (SPS and RDS). Postinterview field notes enhanced this reflective process. NVivo (version 12; QSR International) was used to assist in the organization of interview data and thematic analysis. The team members were in agreement that data saturation occurred at 20 interviews. When using direct quotes from patients, nonidentifiable pseudonyms were used to ensure confidentiality; for example, participant 1, participant 2, and so on.

### Ethical Approval

Ethical approval was obtained from the NHS Health Research Authority and Care Research Wales (reference 19/NE/0318).

## Results

### Overview

A total of 20 participants were recruited and interviewed as part of this study (there were no refusals to partake, participant dropouts, or repeat interviews). Of these 20 participants, 8 (40%) participants were in the preoperative phase and 12 (60%) were in the postoperative phase of their surgical journey. The characteristics of each participant are presented in Table 1. The average age of participants was 46 (SD 10.63) years, and most of the participants had, or were planning to undergo, a gastric bypass procedure (11/20, 55%). All patient interviews were conducted in person between February and March 2020, before the COVID-19 pandemic and restrictions. All participants chose to be interviewed in a confidential room within the bariatric surgery clinic of the hospital. The average interview duration was 52 (SD 18.5) minutes.

The analysis revealed that participants had particular support needs throughout their perioperative journey before and after bariatric surgery. A total of 4 overarching themes were developed from data related to the *capability* and *functionality* of digital health technologies to provide this support. These concerned the technology’s ability to (1) provide surgery-specific content and support; (2) facilitate self-monitoring and goal setting; (3) deliver information in an accessible, trusted, and usable manner; and (4) meet information-seeking and engagement needs at time points before and after undergoing bariatric surgery. We further explored these 4 themes and illustrated perspectives and suggestions with direct interview quotes within this patient-informed piece of work.

### Providing Surgery-Specific Content and Support

When asked about *how* digital technologies could best be designed for patients undergoing bariatric surgery, interviewees expressed opinions about what information should be provided, how this information should be tailored, how specific features could be designed, and their visions of what their *ideal* supportive digital intervention would look like.

It was deemed important that the content and support that patients received from the technology were specific to bariatric surgery. A preoperative participant described how “the support packages should be tailored to the people, rather than the procedure,” explaining how patients “can lose our hair, end up with excess skin, and need to be on lifelong supplements” and how this is “the kind of stuff” that they need support with throughout the journey of surgery and beyond (participant 14, preoperative phase). Another participant explained how it would have been helpful to know that “after a normal operation you’d be able to eat whatever to build up your energy levels again quite quickly...but you can’t do that with bariatric surgery, you physically can’t eat things immediately post-surgery,” so “you’d need it specifically to advise on the bariatric recovery in that case” (participant 3, postoperative phase). There appeared to be an unmet need related to tailored, educational, and informational support for this cohort.

Regarding the content of the technology, discussions centered on dietary-focused forms of support. Patients’ suggestions and desires ranged from the inclusion of “options of what I could have for a snack” (participant 5, preoperative phase) and “something with a meal plan available” (participant 9, preoperative phase), to designing “an app with recipes on it” so patients could “keep coming back to it” for healthier meal options (participant 5, preoperative phase). Patients favored prescriptive approaches (defined as stating what should happen or what someone should do) to content when it came to describing *ideal* technology-enabled support, stating that the intervention should tell them what to do and what to “stick to” (participant 8, postoperative phase). A preoperative patient suggested that the integration of features such as “a list of what you’re not allowed to eat anymore” would be most helpful so they could “easily keep away from it (unhealthy foods)” in a bid to “keep on track” with their anticipated weight loss (participant 14, preoperative phase). In a cohort required to change their lifestyle behaviors, even before undergoing surgery, perhaps technologies delivering short-term descriptive support (defined as describing something in a detailed way) would be beneficial. Participants also stated that immediately following surgery, they wished for stricter prescriptive digital support to help them adjust to their new postoperative lifestyle and dietary intake:

In the first couple of weeks [following surgery], we need to be told what to do, what exactly to do...like what to eat and what to avoid

Participant 9, preoperative phase

Participants considered it important for technology content to also focus on the wider elements of healthy lifestyle behaviors, including increased physical activity and reduced alcohol intake: “If you called it a ‘lifestyles package’ for after bariatric surgery then you can mention things like diet but also [alcohol] drinking and exercise” (participant 9, preoperative phase). Patients demonstrated awareness that positive behavior changes in these areas also contributed to bariatric surgery success, with a participant specifically discussing how they were “trying to look for better choices – like a better choices app” (participant 5, preoperative phase) to support their journey. Interviewees described how building reminders and prompts into technology could better promote these messages of positive health behaviors. The tone and content of these prompts were perceived to be important, combining monitoring and activity messages with motivational statements. The same participant described how patients should be given control over the technology settings so they could decide on the correct tone for them.

I would want something to just give you little reminders – maybe even “have you been weighed this week?” “have you been for a walk?” “don’t let yourself slip”, things like that. But erm, nothing too forceful...Not the whole powered sort of, gym messages, like “get up fatty!” [laughs]

Participant 10, preoperative phase

A postoperative patient reflected that, regardless of the technology delivery method used, “the most important thing is that you’re not left alone after the operation...[as] there’s so many unknowns [sic]” (participant 11, postoperative phase). Instead, they called for tailored digital support to be on hand throughout the entire surgical journey to provide reassurance to patients both pre- and postoperatively.

### Facilitating Self-monitoring and Goal Setting

Both pre- and postoperative participants reflected on the usefulness of self-monitoring and goal-setting functionalities to track their progress throughout the surgical journey. Participants felt it would be useful to self-monitor with “comparison photos” that could be uploaded to an app to “see how much of a difference there has been” (participant 15, postoperative phase). Participants discussed real-time engagement with technologies, remarking the usefulness of inputting daily or weekly weights so that “graphs can track” (participant 7, postoperative phase) and visualize their total weight loss over time. Self-monitoring features were also discussed in association with motivation and emotional investment in the surgical journey, where a participant described how observing “how much [weight] you’ve lost” can “keep people’s spirits up” (participant 15, postoperative phase). Another participant explained how automated messages of “congratulations” were encouraging and “if it calculates your BMI going down as well, I think that would be a really good motivational tool” (participant 7, postoperative phase). Suggestions to incorporate digital self-monitoring features into digital interventions appeared to acknowledge the determination of this cohort in striving for surgical success.

Patients also recognized how self-monitoring could encourage and *push* them to undertake positive health behaviors related to their physical activity to support their postoperative weight loss. A participant described how wearable technology enticed them “into doing more steps or exercise” (participant 1, postoperative phase) and another referred to using gamification features with different *levels* of increased difficulty for them to work through. This participant suggested that increasing the step count targets on a monthly basis would challenge them to continue with regular walking and that achieving the target meant they were encouraged to walk further for the next month. Progressing through these physical activity–based milestones was seen to encourage engagement with their physical rehabilitation and provide underlying reassurance of staying *on track* with their recovery.

I'd want [the physical activity challenges] to have different levels too - like the first month, the second month, unlocking the next bit...Then it’s all there for you and you can keep going back and checking on the app...I can know I’m on track then

Participant 14, preoperative phase

A participant described a common postoperative pitfall of getting “so hung up on what we’re eating and whether it’s right or wrong” (participant 11, postoperative phase). Instead, they recognized the benefit of technological features that enable the setting of “daily goals about exercise” to “give us something else to think about...and work towards” (participant 11, postoperative phase), while achieving their vision of optimal postoperative weight loss. The same participant reflected on how goal setting would have widened their personal knowledge of “what to do after” surgery, meaning they were able to “recover better” (participant 11, postoperative phase) and more successfully. Another participant drew on their personal experiences of using the “NHS Patient Access app” (participant 7, postoperative phase), which is freely available for all patients registered with a general practitioner (physician) in the United Kingdom. The app can be used to view primary care health records, order repeat prescriptions, and get health advice on medical conditions and treatments [56]. This participant suggested that there be inclusion of specialist-bariatric advice within the app, where “the full app [could be linked] to your NHS number so it’s all personalized advice available” (participant 7, postoperative phase). The participant also suggested useful additions to the NHS app, where the home screen could include “tabs at the bottom for specific stuff...like graphs to track [your progress]” (participant 7, postoperative phase).

Participants also discussed the value of shared access to their self-monitored data, where members of the multidisciplinary surgical team were able to track their progress. They remarked that in-built 2-way monitoring features could increase their personal sense of motivation and accountability to “break those [bad] habits” participant 10, preoperative phase), especially knowing that someone else was “keeping an eye” (participant 11, postoperative phase). Another participant felt that shared monitoring could act as a reassurance mechanism for patients, in which they were not being left to “fend for themselves” (participant 4, postoperative phase) in the run-up to surgery or as soon as the surgery was over. A sense of shared responsibility for the success of surgeries was discussed when considering professional-led health care monitoring. A participant supported the inclusion of shared monitoring capabilities so that both patients and health care professionals can “notice if they’re slipping” (participant 16, postoperative phase) off the postsurgical diet, implying that patients alone may not be able to recognize bad habits reforming.

### Delivering Information in an Accessible, Trusted, and Usable Manner

All participants offered suggestions on technology delivery methods and how they would like the intervention to be available to them, including via phone-based apps, web-based forums, and the use of social media platforms such as Facebook. Most participants discussed that their preferred delivery method would be accessible through their smartphone via an app, with a patient explaining “practically everyone knows how to use a phone for stuff now. Everything’s on it...So, if you could put an app on there, I reckon that’s the best way” (participant 15, postoperative phase). Other participants also reported how frequently they used their phones and how people rarely “go anywhere without it,” offering the potential for ongoing engagement even “if I’m out for the day or away on holidays or whatever, I can still log in” (participant 14, preoperative phase) to use it. Many interviewees desired a delivery system that was “nice and clear” (participant 3, postoperative phase), with one remarking they did not want another “dry or crisp NHS website,” instead preferring a “modernized” (participant 4, postoperative phase) app or discussion page.

As an alternative delivery method, some participants reported being members of bariatric surgery groups on Facebook. A few participants reported social media and Facebook to be an acceptable delivery format, offering familiarity and reassurance: ‘I use Facebook all the time...it’s amazing’ (participant 9, preoperative phase). However, participants also questioned the reliability of information posted on Facebook, describing it as “obviously everyone’s own experiences, but it might not necessarily be the safest” (participant 11, postoperative phase). A participant described how some of the posts they had read were “full of nonsense,” and therefore, they got rid of their account. In their view, “an app would be better” as they “would probably trust it [the content] more than Facebook” (participant 5, preoperative phase). Furthermore, another drawback of Facebook was how one “need[ed] to scroll back to find the information,” whereas an app could contain “a specific folder or tab so you could go back to it [information]” (participant 9, preoperative phase). Other participants described their positive experiences of *closed* groups with smaller numbers of individuals. A female patient discussed a private WhatsApp group which contained 5 other postoperative patients and felt that the “how are you all doing? messages” (participant 4, postoperative phase) were helpfully shared among themselves. This indicates that some postoperative patients may find it helpful to surround themselves with smaller groups of like-minded individuals when seeking trusted information.

Many participants highlighted how any information needs to be quick and easy to locate, with one suggesting it should be kept “all together in one place” (participant 9, preoperative phase) and another describing how “that way you can keep coming back to the information any time you wanted to, rather than looking for the leaflets they gave us” (participant 5, preoperative phase). Another described organizing the information with “tabs at the bottom [of the screen] for specific stuff” like “appointments for follow ups” (participant 7, postoperative phase).

Previous technology use was considered along with accessibility and information provision. A participant described “a usable manner” as something that depends “on your character. I’m not very techno-loving or anything, but I’d give it a go [laughs]” (participant 6, postoperative phase). Some participants discussed usability from the perspective of others, particularly the older family members. A interviewee considered her mother aged 63 years, describing how “she can use Google now, but it’s took a long time to get her to do that [sic]. But then again, my husband’s Dad, he’s 73 and he would definitely use digital stuff.” Interestingly, she also appreciated that usability “is a bit dependent on the person too, not just their age” (participant 9, preoperative phase). Some interviewees viewed usability in the same context as familiarity and referred to strategies to overcome this through patient education.

Another participant offered suggestions of how to design the technology so that users of all literary abilities could engage, through the use of *happy* or *sad* faces, or colors, for instance:

I’ve met a lot of people that can’t read or write...you could do happy face, sad face, whatever...Or amber color for not advisable, red for bad or danger, green for good

Participant 12, postoperative phase

### Meeting Patient Information-Seeking and Engagement Needs at Time Points Before and After Surgery

With regard to using a form of digital technology for support, participants shared varying opinions about *when* it would be of most benefit to them during the perioperative period. This benefit appeared to relate to (1) the timing of intervention *implementation* (eg, implementing the technology to enable preoperative information seeking) and (2) the timing of desired *engagement* with technologies (eg, the value of interventions that offered functions that spanned short term and long term to meet patient needs).

When considering their implementation within the surgical journey, participants believed preoperative digital interventions would be useful to acquire knowledge about their upcoming surgery “it’s an operation at the end of the day and you’re changing your insides so I think it’s important to fully know [about] it” (participant 10, preoperative phase). Participants considered this preoperative knowledge-forming period vital for both their physical and mental preparedness. After struggling with their own surgical outcomes, a participant suggested preoperative digital support specifically relating to the psychological preparation of surgery. They discussed how preoperative interventions could better educate patients and meet information-seeking needs and manage postoperative weight loss expectations:

If something could teach me like how to expect, what to expect after [the surgery], it might have helped...“cause I thought the weight loss would be much faster and I look no different now, which has affected my mental health.”

Participant 3, postoperative phase

Similar thoughts were raised by other participants, with one explaining how it “would be really useful to have a map or plan to know what’s going to happen, and when, so we know it’s a full process for us to refer to and not panic” (participant 4, postoperative phase). Another suggested designing “a checklist...like all part of your own bariatric package” where you could “tick off each bit” when it was achieved (participant 3, postoperative phase). Patients may benefit from seeing the phases of the journey and understanding what was going to happen next:

At least you could know what to expect, what is coming either before or after the procedure, and what to do.

Participant 9, preoperative phase

Interviewees recognized the value of information seeking in the initial, short-term, postoperative period “cause, say you were standing in the supermarket and you thought ‘oh I could really fancy that, but I don’t know if I’m allowed it’ then you’d be able to look it up and see if you can have it or not. That would be really practical and handy” (participant 14, preoperative phase). Interviewees recognized that engagement with technologies would likely be higher in the initial postoperative period “once you’ve had it [surgery], you’re in it, and probably will need the information there and then...” (participant 10, preoperative phase), but that each participant’s engagement needs will change, further along their postsurgical surgery they are. Participants also considered the role that technologies could play in terms of long-term ongoing support, where the ability to engage with an intervention, when needed, was deemed important:

It might be something where it [intervention usage] tails off a bit, once you start getting the hang of things, what to eat, how much you can tolerate and stuff. But also, if anything happened and I wanted to ask questions, then I picture being able to use it as and when.

Participant 14, preoperative phase

Two participants (one in the preoperative phase and another in the postoperative phase) acknowledged that technologies could play a role in complementing current practices to improve patient support between annual follow-up appointments. A postoperative participant explained that “once you got a few months in it was more ‘well, I’ll see you in 12 months unless you have problems’ and that's not supportive enough” (participant 11, postoperative phase). They believed there to be benefit from continued technology-enabled engagement throughout this time, specifically linking with a health care professional for advice: “if I’d had more contact with the dietician, digitally, I could maybe have stayed on track better” (participant 11, postoperative phase). Recurring messages of prescriptive and descriptive approaches, in which postoperative participants appear to cede complete control over their journey and outcomes, perhaps demonstrates a lack of belief that they can make and sustain positive behavior changes on their own. A preoperative participant perceived the value of ongoing support from technologies in a more self-determined manner: “I want to make sure I get it [dietary intake] right. I want to avoid any complications and give myself the best chance of success” (participant 5, preoperative phase). They went on to describe their ideal technology-enabled support system, combining technology alongside face-to-face appointments, stating: “I think using tech and still having the [face-to-face] appointments will give me as much support as I need” (participant 5, preoperative phase).

Of all the participants interviewed, only one recommended implementing an intervention that spanned both the pre- and postoperative periods. This patient was in the 2-year postsurgery phase and their views combined those of the pre- and postoperative patients discussed in the previous sections. They described how supportive *boosts* from the technology, continued on a long-term basis, could help to promote positive behaviors:

From the minute you decide to go through with it [surgery], you probably would benefit from having something there just for peace of mind...definitely [implementing] from the start, but also so they can keep using it after [surgery] too for those little boosts and support.

Participant 15, postoperative phase

## Discussion

### Principal Findings

This patient-informed study identified the desires, suggestions, and reflections of bariatric patients in the context of using digital health technologies as support tools during surgery. By collecting both pre- and postoperative patient perspectives, we highlighted *how* digital support strategies could be delivered, *what* content is perceived as useful, and *when* technologies could be implemented within the current NHS bariatric surgery pathway. Our findings discussed 4 key themes related to technology functionality and capability that enable better tailored and targeted digital health technologies for bariatric surgical patients.

### Limitations

Our results have important implications for the design, delivery, usability, and implementation of digital technologies for patients undergoing bariatric surgery. Uniquely, our findings collate participant desires, suggestions, and reflections concerning digital technology use across the entire bariatric perioperative pathway. This study is one of the first to incorporate pre- and postoperative participants, building evidence on the optimization of technology-based support to span the perioperative journey when undergoing bariatric surgery. We acknowledge that there were some limitations to this study. First, the research predominantly focused on a small sample of patients in the North of England. Second, as is common with bariatric surgery, this sample included more female participants than male participants. In addition, we did not assess or sample participants according to their socioeconomic status; it is possible that participants of different socioeconomic classes may have varied experiences with technologies, and our results should, therefore, be interpreted with this in mind. Participants included in this study were purposively sampled from attendees at bariatric surgical clinics (including preoperative assessments and postoperative follow-up appointments); thus, the results do not include patients who were under hospital care but were noncompliant with appointment attendance. Further research that specifically focuses on the experiences and perceptions of participants from ethnic minority communities undergoing bariatric surgery is needed, given that 75% (15/20) of this sample self-reported British or White British ethnicity. Finally, our study also focused solely on the desires, suggestions, and reflections of bariatric surgical patients; thus, the results may not be generalizable to other elective surgical procedures. Future studies may wish to deepen the insights gained from this study to more closely consider patient journey and changing mindsets from pre- to postsurgery phases, which may affect the rates of patient engagement with technologies.

### Comparison With Previous Work

Study participants described a range of potential technological suggestions to meet their pre- and postoperative needs. Patients discussed how digital health technologies could be implemented to enable access to specialist information specific to bariatric surgery, located in an easily accessible place. They demonstrated preferences for digital interventions that incorporated content specific to bariatric surgery rather than being focused on generalized nonsurgical weight loss. Comparable with findings in wider digital health literature, the patients in this study also highlighted the benefits of functionalities that offer support on an individualized basis, such as enabling the provision of individualized feedback and personalized reviews on postoperative progress [57]. Personalization of feedback has previously been associated with positive health behavior changes and increased patient engagement with care [58-60]. A participant suggested connecting technologies to health system identifiers, such as an individual’s NHS number, to support the delivery of personalized care.

In line with this study, perspectives of becoming *digitally engaged patients* were discussed by many participants [61]. For this cohort, the focus of their engagement centered on the monitoring of postoperative progress, primarily the ability to track surgically induced weight loss. Previously, web-based health technologies with monitoring capabilities have been credited as transformers of health care by supporting engaged self-care and promoting positive health behaviors [62]. In addition to individualized feedback, the potential for individualized goal setting may further support the generation of digitally engaged patients with bariatric conditions. Working toward achievable targets has been deemed an effective strategy to successfully motivate behavior change [63]. Wider literature echoes that individualized goal setting has demonstrated improvements in sedentary behavior [64,65], personalized feedback and messages of encouragement have provided patients with cancer, a sense of accomplishment [66], and visual tracking of physical activity (eg, daily step counts) has been reported as motivational [65,67]. Perhaps the same approach could be used for patients undergoing bariatric surgery, with a focus on achievable targets of weight loss, combined with dietary intake and physical activity. Uniquely, a participant reflected on gamification when designing technologies (in game format) to support staged surgical recovery. A study focusing on increased physical activity to aid recovery following cancer surgery expressed similar findings; these authors also identified that personalized difficulty settings in the *game* boosted patient satisfaction and engagement with the intervention [68].

Numerous participants referred to the surgical journey as a process, suggesting that it may benefit from technology-enabled checklists to create a *package of care* between patients and clinicians—a novel finding from this study. Patients envisaged this to be of particular use in the early postoperative period, enabling better control over their recovery and diet and a better understanding of their follow-up care. References were made to design helpful prompts for patients. This echoed previous findings where the tone and delivery of these prompts or messages were deemed crucial in motivating sustained positive health behaviors in patients with cancer [67,69,70].

There appears to be value in implementing technologies both preoperatively and postoperatively. Echoing participant reflections in this study, preoperative interventions have previously been linked with promoting positive behavior change culture [27,28,71]. This is closely linked with theories of surgical teachable moments, arguing that patients are highly susceptible and motivated to change following the initial decision to undergo surgery [72,73]. Highlighting the perspectives of the participants in this study, digital health technologies may present a promising opportunity to prepare patients before surgery and provide continued support between routine postoperative follow-up appointments.

The timing of engagement with technologies appeared to be individualized. The results from this study suggest that, in addition to using technology on a regular basis for personalized prompts and messages, some participants highlighted a desire to engage with the technology on an ongoing basis. The benefit of being able to engage *when required* seems logical, particularly for a patient cohort with changeable postoperative needs over time. The participants in this study also considered that intervention use and engagement rates would likely be higher soon after surgery but reduce over time once they better adjusted to their life after surgery. The dichotomy concerning intervention timings revealed in this study draws attention to the importance of finding optimal *engagement balance* with any digital health technologies implemented for patients. Currently, there is insufficient evidence to state the optimal initiation point and ongoing engagement points of digital technologies within the bariatric surgical pathway, an area that future studies may explore further.

Participants raised contrasting views that suggested a fine balance existed between them accepting and abdicating responsibility over their recovery and subsequent surgical *success*. Prescriptive and descriptive approaches to technology content were desired by some who wanted the technology to provide them with regulated and specific advice, such as directed postoperative meal plans. However, previous studies have noted this approach to have questionable success when it comes to motivating and sustaining behavior change [74]; instead, the authors have cited the importance of empowered patient–health provider strategies [75,76]. Self-determination Theory (SDT) provides a theoretical framework to understand participant motivations and behaviors [77]. When SDT was applied to other health behavior contexts (such as programs for smoking cessation [78] and weight loss [79]), findings suggested that the more autonomously motivated participants were, the more successfully they implemented behavior change. The information-motivation-behavioral skills model of health behavior has been widely used in medical research [80-82] to understand and improve patient health behaviors and increase the efficacy and effectiveness of behavioral interventions. This model states that educational information (which could be prescriptive in nature, as desired by this cohort) is a prerequisite to successfully enact a change in health behaviors [83]. Both the SDT and information-motivation-behavioral skill models propose that patients who are well educated and informed, with higher levels of independent motivation and acceptance of responsibility, are more likely to enact and maintain health-related behaviors. In the context of this study, the desire for prescriptive and descriptive approaches to technology content is not necessarily at odds with the need for interventions that boost patient motivation; both approaches may be regarded as requirements for supporting successful patient weight loss, both in the short term and long term.

Technology-enabled monitoring has also been recognized to boost autonomous motivational levels [77]; however, long-term monitoring by health care professionals as desired by the patients may be considered unsustainable. Monitoring opportunities and timescales should be considered when it comes to digital technology design and functionality to support and motivate patients during their surgical journey. Given its value as a source of potential accountability and motivation for self-monitoring and social support benefits, digitally-enabled peer networking within the bariatric surgical journey should be considered as an area for future research, in particular, *how* and *when* digital health technologies could support with, and facilitate, this [25]. Future research should focus on the motivational role of digital technologies when providing support to patients facing challenges within the bariatric surgical pathway, such as regaining weight.

Similar to previous digital health research, themes of usability were discussed by participants, particularly regarding their existing familiarity versus unfamiliarity with technologies [84]. Reflections from the perspective of older relatives highlighted that digital literacy and generational bias may still be a challenge to overcome when considering the implementation of health technologies [67,85,86]. Although technologies are now implemented more readily within health care, some patients may still prefer face-to-face encounters with clinicians rather than web-based ones [61]. We should be mindful of acknowledging this and, as suggested by the participants, work to complement technological integration with educational support materials.

### Conclusions

Perceptions of patients undergoing bariatric surgery validate the integration of digital health technologies within the surgical care pathway, offering enhanced connectedness and support. The findings from this study have the potential to influence the design and targeting of future digital technologies to best support bariatric surgical patients. To achieve surgical success, digital strategies should consider the incorporation of specialist information tailored to the bariatric surgery cohort and the implementation of self-monitoring and goal-setting functionalities at various time points within the bariatric surgical pathway. Further, to address the specific unmet support needs of this patient cohort, digital health technologies should enable the provision of a *package of care* to offer long-term lifestyle support.

## Appendix

Multimedia Appendix 1 Qualitative research checklist and interview topic guide.

## Abbreviations

COREQ: Consolidated Criteria for Reporting Qualitative Research
NHS: National Health Service
SDT: Self-determination Theory
